# Neuroprotective Effect of Cardamom Oil Against Aluminum Induced Neurotoxicity in Rats

**DOI:** 10.3389/fneur.2019.00399

**Published:** 2019-04-30

**Authors:** Sandip T. Auti, Yogesh A. Kulkarni

**Affiliations:** Shobhaben Pratapbhai Patel School of Pharmacy & Technology Management, SVKM's NMIMS, Mumbai, India

**Keywords:** Alzheimer's disease, acetylcholinesterase, aluminum, cardamom oil, oxidative stress, neuroprotective

## Abstract

Acetylcholinesterase (AChE) is an enzyme involved in the progression of Alzheimer's disease (AD). Cardamom oil (CO) has been reported to have acetylcholinesterase inhibitory, antioxidant and anti-anxiety effects. Hence, we studied the effect of cardamom oil in aluminum chloride induced neurotoxicity in rats. AD like symptoms were induced in Wistar rats with aluminum chloride (100 mg/kg, *p.o*.). Cardamom oil was administered concomitantly by oral route at doses of 100 and 200 mg/kg for 42 days. Behavioral parameters like Morris water maze, elevated plus maze, passive avoidance test and locomotor activity were evaluated on day 21 and 42. AChE activity, oxidative stress parameters, histopathological studies and immunohistochemistry studies were carried out in hippocampus and cortex. Cardamom oil treatment showed significant improvement in behavioral parameters, inhibition of AChE activity (*p* < 0.001) and reduction in oxidative stress in the brain. Histopathological studies of hippocampus and cortex by hematoxylin & eosin (H. & E.) and congo red stain showed inhibition of neuronal damage and amyloid β plaque formation with cardamom oil treatment. Immunohistochemistry showed, CO treatment inhibited amyloid β expression and upregulated brain-derived neurotrophic factor (BDNF). The present study showed that, cardamom oil has neuroprotective effect in aluminum chloride induced neurotoxicity linked with inhibition of AChE activity and reduction in oxidative damage. This effect of cardamom oil may be useful in management of Alzheimer's disease.

## Introduction

Alzheimer's disease (AD) is an age-associated progressive neurodegenerative disorder. It is clinically characterized by a decline in cognitive function and pathologically defined by the accumulation of intracellular neurofibrillary tangles (NFTs) and extracellular β-amyloid (Aβ) plaques in the brain (1). Deposition of β-amyloid protein results into formation of Aβ plaques and hyperphosphorylation of tau protein aggregates into neurofibrillary tangles. The major symptoms of AD include loss of cognitive functions and motor activities (1, 2). Patients with AD have cholinergic deficits, which induces learning and memory impairments (3, 4). Environmental factors increase the risk of AD such as exposure to metals. Heavy metals like aluminum and iron mainly affect brain development and are reported to the main progenitor in several neurodegenerative disorders such as Parkinson's disease and Alzheimer's disease (2). Exposure to aluminum causes activation of AChE enzyme which is involved in the pathogenesis of AD (5). Several characteristic factors such as an increase in oxidative stress, acetylcholinesterase (AChE) activity, aggregation of Aβ plaques, tau protein and decrease in acetylcholine (ACh), brain-derived neurotrophic factor (BDNF) in the brain are involved in etiology of AD (5). In Alzheimer's disease, the increased activity of the AChE leads to the breakdown of the ACh that causes depletion of acetylcholine level, resulting into impaired cholinergic functions in the brain (6). It has been shown that, AChE promotes the aggregation and formation of Aβ fragments in the brain (7). It has been reported that AChE inhibitors such as donepezil hydrochloride enhance neurogenesis in the brain by decreasing AChE and increasing BDNF (8, 9). BDNF is one of the most widely distributed neurotrophins present in the brain. BDNF plays an important role in neurogenesis, neuronal plasticity, neuronal survival, serves as a neurotransmitter modulator and is widely expressed in the brain (10). It also participates in Long Term Potentiation (LTP), an essential process involved in learning and memory (11). It has been reported that patients with certain neurologic disorders, such as Alzheimer's disease (12, 13) and Parkinson's disease (14) contain high concentration of aluminum in their autopsied brain samples. It has been reported that elevated level of aluminum specifically in the hippocampus part of a brain lead to development of amyloid beta plaques, neurofibrillay tangles in AD patients (15).

Current pharmacotherapy for AD provides only symptomatic relief. Acetylcholinesterase (AChE) inhibitors e.g., galantamine, rivastigmine, donepezil hydrochloride, which produces side effects like, bronchoconstriction and hypotension and NMDA (N-methyl-D-aspartate) receptor antagonist e.g., memantine produces side-effects like, nausea, vomiting, and diarrhea.(16, 17) So, there is an urgent need for development of new drugs for treatment or management of AD. Now, global therapeutic research is shifting toward herbal or alternative system of medicine. Natural products have reported for their neuroprotective action in various disease pathways which are involved in the progression of AD (18). Herbal drugs are popular because people presumes that herbs are safer than synthetic drugs, though they should be used with caution as synthetic active products. Terpenoids are mixture of naturally occuring, volatile compounds which are synthesized by plants in the form of secondary metabolites. The essential oils (EOs) obtained from certain plants such as juniper oil from common juniper (*Juniperus communis* L) (19) and clove oil obtained from clove buds (*Eugenia caryophyllus*) (20) are reported to have anticholinesterase activity (8, 21, 22). So, essential oils may show their neuroprotective effect by down-regulation of AChE and up-regulation of BDNF in the brain.

In traditional medicine, use of cardamom, *Elettaria cardamomum* L. (Zingiberaceae) has been indicated in various medical problems such as anxiety, convulsion, insomnia, loss of appetite due to its medicinal importance. The fresh fruits of cardamom are routinely added in various dishes to impart delicious taste and flavor in Asian countries. The major chemical constituents of cardamom oil are 1,8 cineole (25–45%), α-terpinyl acetate (20–53%), limonene (5.6%), linalyl acetate (8.2%), and linalool (5.4%) (23). Cardamom oil has been shown to exhibit wide variety of pharmacological and biological activities namely, anti-convulsant (24), gastro- protective (25), antioxidant (26), anti-inflammatory (27), antimicrobial (28), chemopreventive (29) anti-anxiety (30), antihyper-cholesterolemic (31). The major phytoconstituent of cardamom oil, 1,8 cineole showed AChE inhibitory activity (32). Based on this background, the present study was designed to explore the neuroprotective effect of cardamom oil in aluminum chloride induced oxidative damage and cognitive impairments in rats.

## Materials and Methods

### Drugs and Chemicals

Aluminum chloride, acetylthiocholine iodide was procured from Sigma Aldrich (St. Louis, MO, USA). β-Amyloid (B-4) antibody, mouse anti-rabbit-IgG-HRP antibody, pro BDNF (5H8) antibody were purchased from Santa Cruz Biotechnology, Inc., USA. Cardamom oil was purchased from iFRAGRANCE INDIA, Kannauj, India. Donepezil hydrochloride was obtained from Micro Labs Limited, Mumbai, India as a gift sample. All other chemicals used were of analytical grade.

### Volatile Oil Analysis

Cardamom oil was analyzed by GC-MS using Agilent 6890 N Network GC system. Capillary column used was BPX 35 (30 m × 0.25 mm; film thickness 0.25 μm) coated with 35% Phenyl Polysilphenylene-siloxane using helium as a carrier gas (1.0 ml/min). The temperature programme started with 50°C to 220°C at a rate of 10°C per minute, split ratio was kept 1:25 and flame ionization detector (FID) was used during analysis. The acquisition mass range was kept 40–400 amu. Two percent solution of cardamom oil was prepared in ethanol (95%) and two replicates of the samples processed in the same way. The injection volume was kept 1.0 μl. The identification of the compounds from the oil sample was done and compared them with respect to their retention times (RT) and mass spectra that obtained from authentic Wiley libraries (available through Hewlett Packard) and the literature (33).

#### Animals

Male Albino Wistar rats (180–220 g) were procured from National Institute of Biosciences, Pune, India. Animals were maintained at 12/12 hr light/dark cycle at room temperature of 25 ± 2°C, relative humidity of 40–60%. Animals were acclimatized for seven days prior to the experiment. Animals had access to standard pellet diet and water *ad libitum*. The study protocol was approved through Institutional Animal Ethics Committee (approval no. CPCSEA/IAEC/P-24/2018 dated 18^th^ April 2018) which was formed in accordance with the norms of the Committee for the Purpose of Control and Supervision of Experiments on Animals (CPCSEA), Government of India and complied with the National Institutes of Health (NIH) guidelines on handling of experimental animals.

### Experimental Design

Aluminum chloride solution was prepared in distilled water; cardamom oil and Donepezil hydrochloride were suspended in 5% Tween 80. Sixty male albino Wistar rats (180–220 g) were randomized based on their body weight into five groups of 12 animals each. The group I was kept as normal control and received 5% Tween 80 orally. The other groups orally received aluminum chloride solution (100 mg/kg) for 42 days (34). Group II was kept as disease control, Group III was kept as a standard which received Donepezil hydrochloride (1 mg/kg) orally 1 h after aluminum chloride administration. Group IV and V received cardamom oil at dose 100 and 200 mg/kg orally respectively, for 42 days, 1 h after aluminum chloride administration.

### Behavioral Assessment

#### Locomotor Activity

Locomotor activity was performed on day 21 and 42 of aluminum chloride administration. Each animal was kept in digital actophotometer and observed for 5 min, the apparatus equipped with infrared light sensitive photocells. When the beam of light falling on the photo cell is cut off by the animal that considered as one count and was recorded, values were expressed as number of counts per 5 min (34).

#### Morris Water Maze (MWM)

MWM test is used to assess spatial memory task in rodents. It was performed as per earlier method described (34, 35) with slight modifications. A large circular swimming tank (150 cm diameter, 45 cm height) consisted of four equal quadrants (NW, NE, SE, and SW) containing water (25 ± 1°C) was used. Visual cues in the form of red and blue colored tapes were placed around the water tank for facilitation of the spatial orientation in experimental animals; positions of the cues were kept unchanged throughout the experiment. The submerged platform (10 × 10 cm), was kept 1 cm above the surface of water in the acquisition phase. During the acquisition phase, the animal was placed in the tank, facing toward the wall of tank and allowed for 120 s to locate the platform. The animal was guided to reach the platform, if failed within 120 s and placed for 30 s on the platform. Daily four trials were given to each animal for four consecutive days (17^th^ to 20^th^ day) at the interval of 10 min maintained between each trial. Animal was gently placed in different quadrants of swimming tank during each trial. During the retention phase, water surface made opaque by using milk powder in order to hide the platform and kept 1 cm below the level of water in the tank. The animal was placed in the quadrant of tank facing toward the wall of the tank and retention of memory of the animal was evaluated on day 21 and 42. Escape latency was calculated by measuring time taken to locate the hidden platform by the animal in water maze.

#### Elevated Plus Maze (EPM)

EPM test is used to assess anxiety in rodents and was performed as per earlier method described (34, 36). The elevated plus maze apparatus consisted of two closed walls (50 × 10 cm), transverse with two open arms with same dimensions each and having 40 cm high walls. Both the arms of EPM were connected with central square (10 × 10 cm) and maintained 50 cm height above the ground level. During the acquisition phase, the animal was placed at one end of arm, facing away from central square area. Initial transfer latency (ITL) was recorded as the time required to the animal to move from open arm to closed arm. After recording ITL, each animal was allowed to explore for 20 s inside the maze and after that returned to the home cage. Retention of memory was evaluated after 24 h by placing the animal in an open arm and the 1^st^ transfer latency and 2^nd^ transfer latency was recorded on day 21 and 42, respectively.

#### Passive Avoidance Test (PA)

PA test is used to assess memory retention deficit in rodents and was performed as per earlier method described (37). The apparatus consisted of both light and dark compartment having dimensions (25 × 25 × 25 cm) of each, illuminated with a 40W bulb and a dark chamber, separated by a guillotine door. The bottom of the dark compartment equipped with a metal grid floor with a shock generator to induce electric stimuli. In the acquisition trial, the animal was placed in the light compartment and kept inside for 60 s for habituation. After this guillotine door was opened and the latency to step into dark compartment was recorded as pre-shock latency. Once the animal entered in the dark compartment, a mild electric stimulus of 0.5 mA for 2 s period was given through the grid floor and then taken out. The animal was immediately removed from the compartment and returned to the home cage. After 24 h of acquisition trial, retention trial was performed in which no electric stimulus was given when rat entered in the dark compartment. Time taken to step into dark compartment was recorded as post-shock latency in seconds.

### Biochemical Assessment

#### Collection of Brain Tissues

At the end of the behavioral studies, animals were sacrificed and brain tissues were collected, rinsed with ice-cold isotonic saline solution and stored at −80°C.

#### Assessment of Oxidative Stress Parameters

Hippocampus and cortex part of the brain were excised and homogenized in 10 volume of ice-cold 0.1M phosphate buffer solution (pH 7.4) using probe homogenizer (Polytron PT 2500E, Kinematica, Switzerland). Total protein content was determined in as such homogenate as the method described by Lowry et al. (38), MDA level in hippocampus and cortex were determined by using the method described by Ohkawa et al. (39), SOD assay was measured in post-mitochondrial supernatant according to the method described by Paoletti et al. (40, 41), post-nuclear supernatant obtained from homogenate was used to perform catalase assay as method described by Lück (42), Reduced glutathione level in hippocampus and cortex was measured according to method of Ellman (43).

#### Acetylcholinesterase Activity (AChE)

The acetylcholinesterase activity in brain tissue was performed according to the method described by Ellman et al. (44) with slight modifications. The assay mixture contained 0.1 ml of supernatant, 2 ml of sodium phosphate buffer (0.1M, pH 8.0) containing 0.1% BSA, 0.1 ml of dithio-bis-nitrobenzoic acid (DTNB) and 0.05 ml of acetylthiocholine iodide (AChI). The change in absorbance was measured for 2 min at 1 min interval at 412 nm using UV-VIS Spectrophotometer, (Perkin Elmer Lambda 20, USA). acetylcholinesterase activity was expressed as micromoles of acetylthiocholine iodide hydrolyzed per min per mg protein.

### Histopathological Studies

Brain tissues stored in 10% neutral buffered formalin (NBF) (4 g of sodium phosphate monobasic anhydrous, 8.15 g sodium phosphate dibasic dihydrate, 100 ml formalin, and 900 ml distilled water) were used for histopathology study. It was fixed in paraffin blocks and thin transverse sections of 5 μm thickness were taken using microtome (Leica, USA) of hippocampus and cortex region. The sections were stained with hematoxylin and eosin (H&E), Congo red dye and examined under digital microscope (Motic, Canada). The observations of the sections were carried out at 400X. For histology analysis six slides per group were evaluated for cortical and hippocampal region (complete section). Morphometric analysis was carried out for evaluation of the extent of neurodegeneration and amyloid beta deposition in brain (45, 46) and severity index was considered as No Abnormality Detected, Minimal (<1%), Mild (1–25%), Moderate (26–50%), Marked/Moderately Severe (51–75%), Severe (76–100%), and distribution was recorded as focal, multifocal and diffuse. Optical density (OD) was determined to measure the extent of amyloid β plaques deposited in hippocampus and cortex in congo red staining by analyzing the images with Image J 1.51a software (NIH, USA).

### Immunohistochemistry for Determination of Amyloid β and BDNF Expression in Brain Tissue

Amyloid β and BDNF expression in hippocampus and cortex were carried out by immunohistochemical staining as per earlier method described (45, 47, 48). Paraffin wax embedded tissue blocks were cut and transverse sections at 5–6 μm thickness were taken with the microtome (Leica, USA) and placed on slides coated with Poly-L-Lysine and placed in an incubator overnight at 37°C for 1 h. Further these sections were deparaffinized, rehydrated and incubated with citrate buffer, pH 6 at decloaking chamber. Slides were incubated in 3% hydrogen peroxide block for 20 min to block endogenous peroxidase. β-Amyloid (B-4) antibody and pro BDNF (5H8) antibody (Santa Cruz Biotechnology, Inc., USA) were applied as the primary antibody and secondary antibody like mouse anti-rabbit-IgG-HRP (Santa Cruz Biotechnology, Inc., USA) was used. The staining was visualized by reaction with diaminobenzidine (DAB) reagent and further counterstained with hematoxylin. The sections were rinsed with Tris buffer saline (TBS) and dehydrated in alcohol and cleared in xylene prior to mounting using DPX. All the sections were examined under the digital microscope (Motic, Canada) to record the intensity of antigen antibody reaction. The observation of the sections was carried out at 400X. For immunohistochemistry analysis six slides per group were evaluated for hippocampal and cortical region (complete section). Morphometric analysis was carried to observe extent of immunoreactivity. Optical density (OD) was determined as measure of amyloid β and BDNF expression in hippocampus and cortex by analyzing the images with ImageJ 1.51a software (NIH, USA).

### Human Equivalent Dose Calculation

Cardamom oil is generally recognized as safe (GRAS) and is listed by Flavor and Extract Manufacturers Association (FEMA) number- 2241. Human Equivalent Dose (HED) can be calculated from animal dose based on body surface area. For conversion of animal dose to HED, animal dose is multiplied by K_m_ ratio. K_m_ is correction factor estimated by dividing the average body weight (kg) of species to its body surface area (m^2^). K_m_ ratio for animal to human is 0.16 (49)

$$\begin{array}{l} \text{HED }\left(\text{mg}/\text{kg}\right)=\text{Animal dose }\left(\text{mg}/\text{kg}\right)\times \;0.16 \end{array}$$

### Statistical Analysis

The differences among treatment and control groups were analyzed by using GraphPad Prism V_5.0_ for Windows. Behavioral parameters were assessed by two-way ANOVA (analysis of variance) followed by Bonferroni's test and the significance level was determined within and between groups. Oxidative stress parameters, AChE activity and immunohistochemistry quantification data were assessed by one-way ANOVA followed by Dunnett's multiple comparison test. All data are expressed as mean ± standard error of mean (SEM); *p* < 0.05 were considered to be significant.

## Results

### Chemical Composition of the Cardamom Oil

The chemical composition of the cardamom oil was analyzed by GC–MS/FID. The identity of main constituents was confirmed by their mass fragmentation analysis. The main compounds in oil identified were: Terpinyl acetate, 1,8 Cineole, α-terpineol, linalool, followed by lower quantities of trans-geraniol, L-4 - terpineol, D-limonene, linalyl anthranilate, β-pinene (Table 1).

**Table 1:** Phytochemical composition of cardamom oil by GC-MS.

**Sr. no**.	**Compound**	**Retention time (min)**	**Match factor**	**Area (%)**
1	D-limonene	7.81	925	2.96
2	1,8 Cineole	7.90	956	31.09
3	Linalool	9.18	943	5.73
4	L-4 - terpineol	10.83	928	3.13
5	α-terpineol	11.12	929	6.51
6	linalyl anthranilate	11.85	949	2.35
7	trans-geraniol	11.99	925	3.17
8	Terpinyl acetate	13.68	948	42.91
9	β-pinene	14.09	920	2.11

### Behavioral Assesment

#### Locomotor Activity

**Figure 1:**
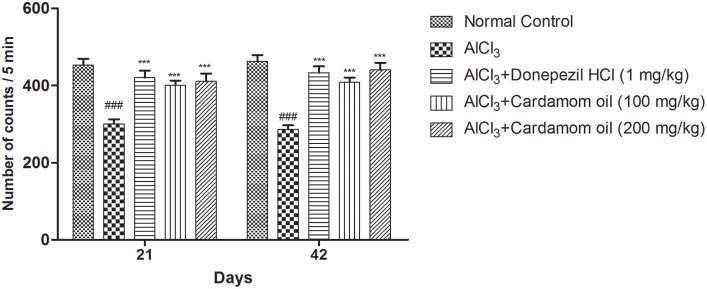
Effect of Cardamom oil on locomotor activity of rats using actophotometer on AlCl_3_-induced neurotoxicity. Data are expressed as mean ± SEM (*n* = 6), ###*p* < 0.001 when compared with normal control, ^***^P < 0.001 when compared with disease control group. AlCl_3_, Aluminum chloride; SEM, Standard Error of Mean.

The locomotor activity was significantly decreased in the aluminum chloride treated group when compared with the normal control group. Cardamom oil treatment at both selected dose levels significantly improved locomotor activity on day 21 and 42 (*p* < 0.001) when compared with the disease control group indicating improvement in aluminum chloride induced impaired locomotion. The cardamom oil treatment showed comparable results as that of donepezil hydrochloride treated group (Figure 1).

#### Morris Water Maze

**Figure 2:**
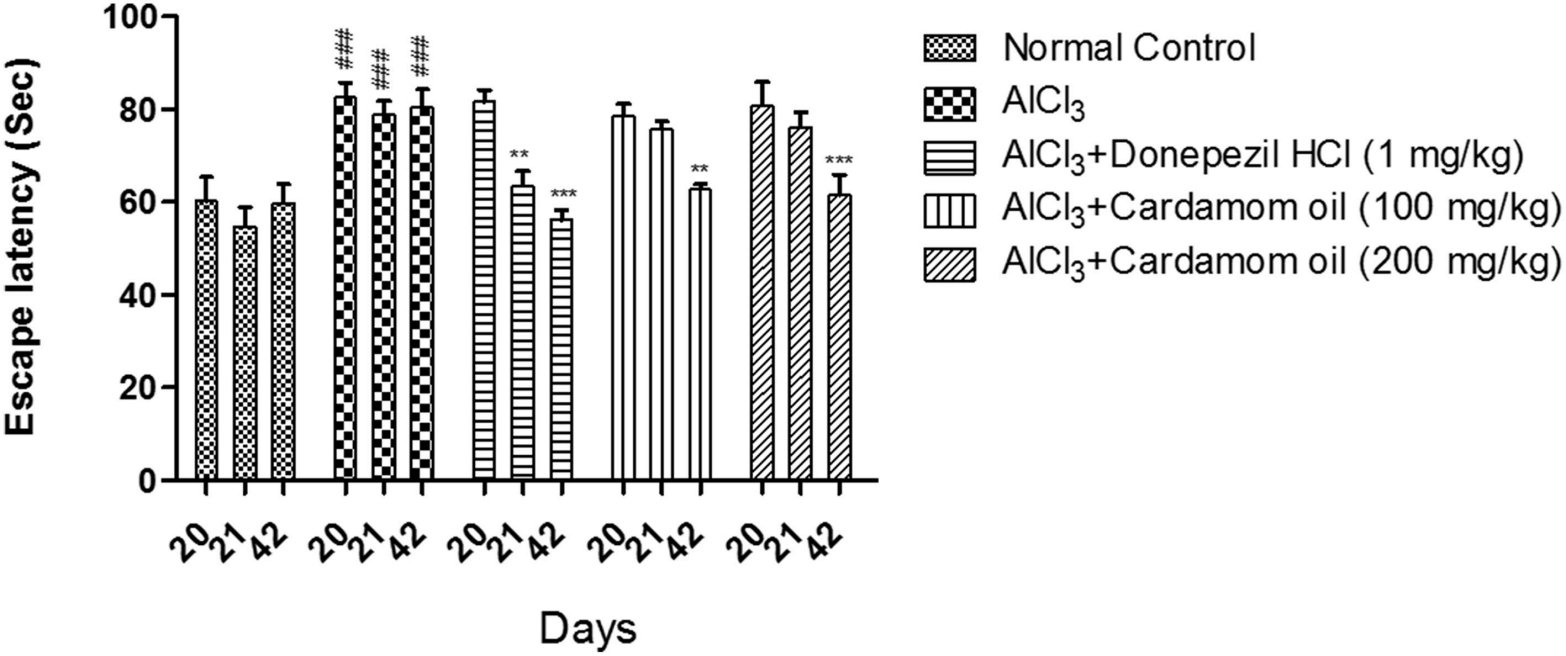
Effect of Cardamom oil on Morris water maze test in AlCl_3_-induced neurotoxicity in rats. Data are expressed as mean ± SEM (*n* = 6), ###*p* < 0.001 when compared with normal control, ^**^p < 0.01, ^***^P < 0.001 when compared with disease control group. AlCl_3_, Aluminum chloride; SEM, Standard Error of Mean.

In the Morris water maze test, the aluminum chloride treated group showed a significant increase in escape latency when compared with normal control group. However, cardamom oil treatment at a dose of 100 and 200 mg/kg significantly prevented the increase in escape latency produced by aluminum chloride treatment on day 42 (*p* < 0.001) when compared with disease control group. The cardamom oil treatment showed comparable results as that of donepezil hydrochloride treated group and improved the retention performance of the spatial navigation task (Figure 2).

#### Elevated Plus Maze

**Figure 3:**
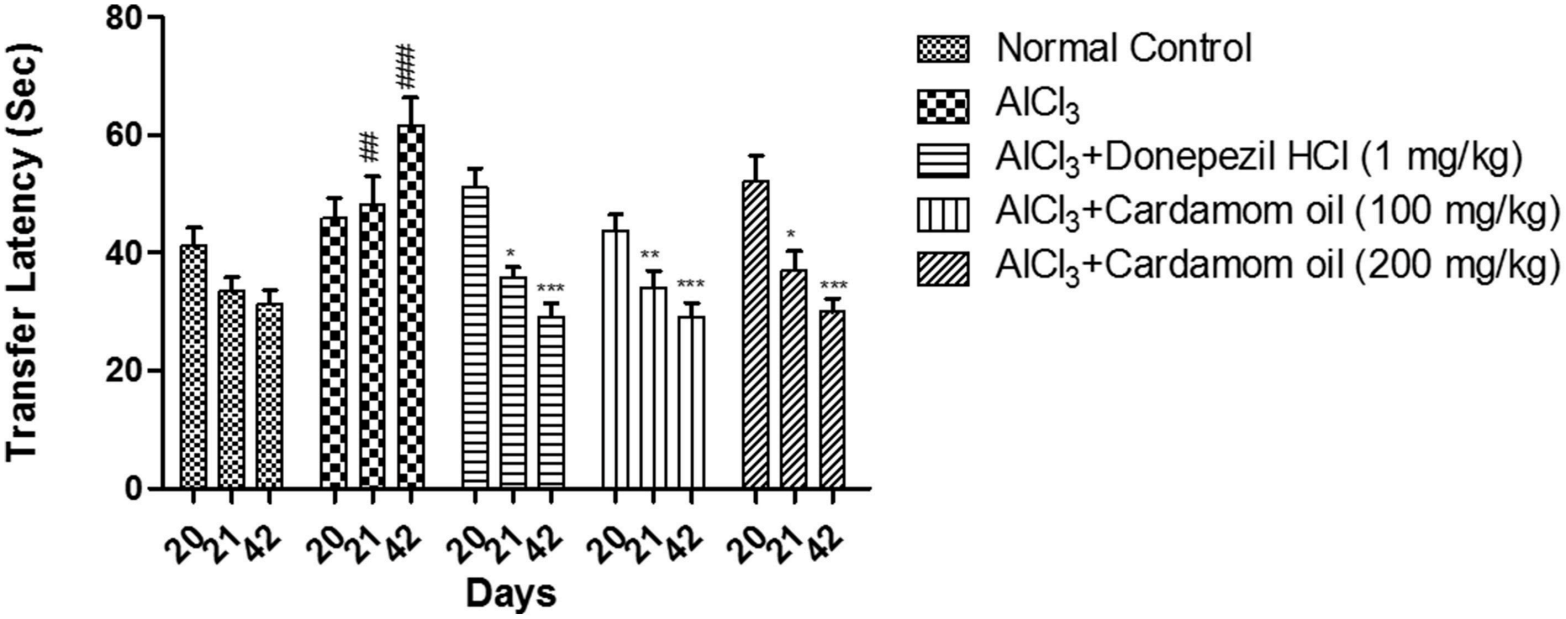
Effect of Cardamom oil on transfer latency of rats using Elevated plus maze test in AlCl_3_-induced neurotoxicity. Data are expressed as mean ± SEM (*n* = 6), ##p < 0.01, ###*p* < 0.001 when compared with normal control, ^*^p < 0.05, ^**^p < 0.01, ^***^P < 0.001 when compared with disease control group. AlCl_3_, Aluminum chloride; SEM, Standard Error of Mean.

In elevated plus maze test, the aluminum chloride treated group showed significant increase in 1^st^ transfer latency and 2^nd^ transfer latency at day 21 and 42 respectively, with respect to initial transfer latency (ITL) at day 20, when compared with normal control group. Cardamom oil treatment at a dose of 100 and 200 mg/kg significantly prevented aluminum chloride induced increase in 1^st^ transfer latency and 2^nd^ transfer latency on day 21 (*p* < 0.01) and 42 (*p* < 0.001) when compared with disease control group. The cardamom oil treatment showed comparable results as that of donepezil hydrochloride treated group and improved the memory performance in animals (Figure 3).

#### Passive Avoidance Test

**Figure 4:**
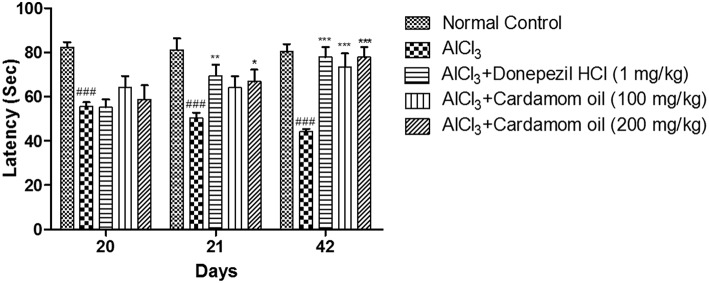
Effect of Cardamom oil on latency of rats using Passive Avoidance test in AlCl_3_-induced neurotoxicity. Data are expressed as mean ± SEM (*n* = 6), ###*p* < 0.001 when compared with normal control, ^*^p < 0.05, ^**^p < 0.01, ^***^P < 0.001 when compared with disease control group. AlCl_3_, Aluminum chloride; SEM, Standard Error of Mean.

In the passive avoidance test, the escape latency (EL) was measured on day 20 and the retention latency (RL) was measured on 21^st^ and 42^nd^ day, respectively. In aluminum chloride treated group, a significant decrease in RL was reported as compared to normal control group. Treatment with cardamom oil at a dose of 200 mg/kg showed significant recovery in retention latency on day 42 (*p* < 0.001) when compared with the disease control group. Treatment with donepezil hydrochloride at a dose of 1 mg/kg showed more significant results on day 21 (*p* < 0.01) than cardamom oil treatment, while the level of significance was similar for these groups on day 42 (*p* < 0.001) (Figure 4).

### Assessment of Oxidative Stress Parameters

#### Assessment of Oxidative Stress Parameters in Hippocampus

Aluminum chloride treated animals showed a significant increase in MDA level (*p* < 0.001) and significant decrease in level of GSH (*p* < 0.01), SOD (*p* < 0.01) and catalase activity (*p* < 0.01) in hippocampus when compared with normal control animals. MDA level was significantly reduced in cardamom oil treatment at a dose of 100 and 200 mg/kg (*p* < 0.001) when compared with the disease control group. Cardamom oil treatment showed significant improvement in GSH and SOD levels at a dose of 200 mg/kg (*p* < 0.01) as compared with disease control group. Treatment with cardamom oil at a dose of 200 mg/kg (*p* < 0.05) significantly improved catalase activity as compared with disease control group (Table 2).

**Table 2:** Effect of Cardamom oil on brain oxidative stress parameters in hippocampus.

**Group**	**MDA (nmol mg protein ^**−1**^)**	**GSH (μmol mg protein ^**−1**^)**	SOD (U mg protein^−1^*^^100)	**CAT (nmol of H_**2**_O_**2**_ decomposed min^**−1**^ mg protein)**
Normal control	4.07 ± 0.3400	10.41 ± 0.5244	8.30 ± 0.4	7.5 ± 0.6
AlCl_3_	6.62 ± 0.7810^###^	5.84 ± 0.3428^##^	5.26 ± 0.4^##^	4.2 ± 0.4 ^##^
AlCl_3_ + Donepezil HCl (1mg/kg)	3.92 ± 0.2393^***^	8.30 ± 0.5127^**^	7.80 ± 0.55^**^	7.0 ± 0.9^**^
AlCl_3_ + Cardamom oil (100 mg/kg)	4.02 ± 0.4214^***^	7.60 ± 0.5244	6.57 ± 0.68	5.6 ± 0.6
AlCl_3_ + Cardamom oil (200 mg/kg)	3.84± 0.3326^***^	8.36 ± 0.5368^**^	7.93 ± 0.36^**^	6.3 ± 0.4^*^

*Data are expressed as mean ± S. E. M. (n = 6)*,

### *p < 0.001*,

## *p < 0.01 when compared with normal control*,

*** *p < 0.001*,

** *p < 0.01*,

* *p < 0.05 when compared with disease control group*.

#### Assessment of Oxidative Stress Parameters in Cortex

Cortex region showed a significant increase in MDA level (*p* < 0.001) and significant decrease in level of GSH (*p* < 0.001), SOD (*p* < 0.001), and catalase activity (*p* < 0.001) in aluminum chloride treated animals when compared with the normal control. MDA level was significantly reduced in cardamom oil treatment at dose of 100 mg/kg (*p* < 0.05) and 200 mg/kg (*p* < 0.001) when compared with the disease control group. Treatment with cardamom oil significantly ameliorated GSH level at a dose of 100 mg/kg (*p* < 0.05) and 200 mg/kg (*p* < 0.001) when compared with disease control group. SOD level was significantly improved in cardamom oil treatment at dose of 100 mg/kg (*p* < 0.05) and 200 mg/kg (*p* < 0.01) when compared with disease control group. Catalase activity was significantly improved in cardamom oil treatment at a dose of 200 mg/kg (*p* < 0.01) when compared with the disease control group. The cardamom oil treatment showed comparable results as that of donepezil hydrochloride treated group (Table 3).

**Table 3:** Effect of Cardamom oil on brain oxidative stress parameters in cortex.

**Group**	**MDA (nmol mg protein^**−1**^)**	**GSH (μmol mg protein^**−1**^)**	SOD (U mg protein^−1^*^^100)	**CAT (nmol of H_**2**_O_**2**_ decomposed min^**−1**^ mg protein)**
Normal control	3.924 ± 0.5230	11.050 ± 0.6864	8.94 ± 0.66	8.2 ± 0.3
AlCl_3_	6.793 ± 0.6333^###^	4.643 ± 0.4799^###^	4.70 ± 0.47^###^	3.7 ± 0.5^###^
AlCl_3_ + Donepezil HCl (1mg/kg)	3.143 ± 0.3242^***^	7.140 ± 0.6050^*^	7.56 ± 0.35^**^	9.1 ± 0.7^***^
AlCl_3_ + Cardamom oil (100 mg/kg)	4.994 ± 0.4586^*^	6.854 ± 0.4486^*^	7.40 ± 0.65^*^	5.0 ± 0.8
AlCl_3_ + Cardamom oil (200 mg/kg)	3.134 ± 0.1432^***^	8.159 ± 0.5251^***^	7.61 ± 0.54^**^	6.8 ± 0.5^**^

*Data are expressed as mean ± S. E. M. (n = 6)*,

### *p < 0.001 when compared with normal control*,

*** *p < 0.001*,

** *p < 0.01*,

* *p < 0.05 when compared with disease control group*.

### Acetylcholinesterase Assay (AChE)

**Figure 5:**
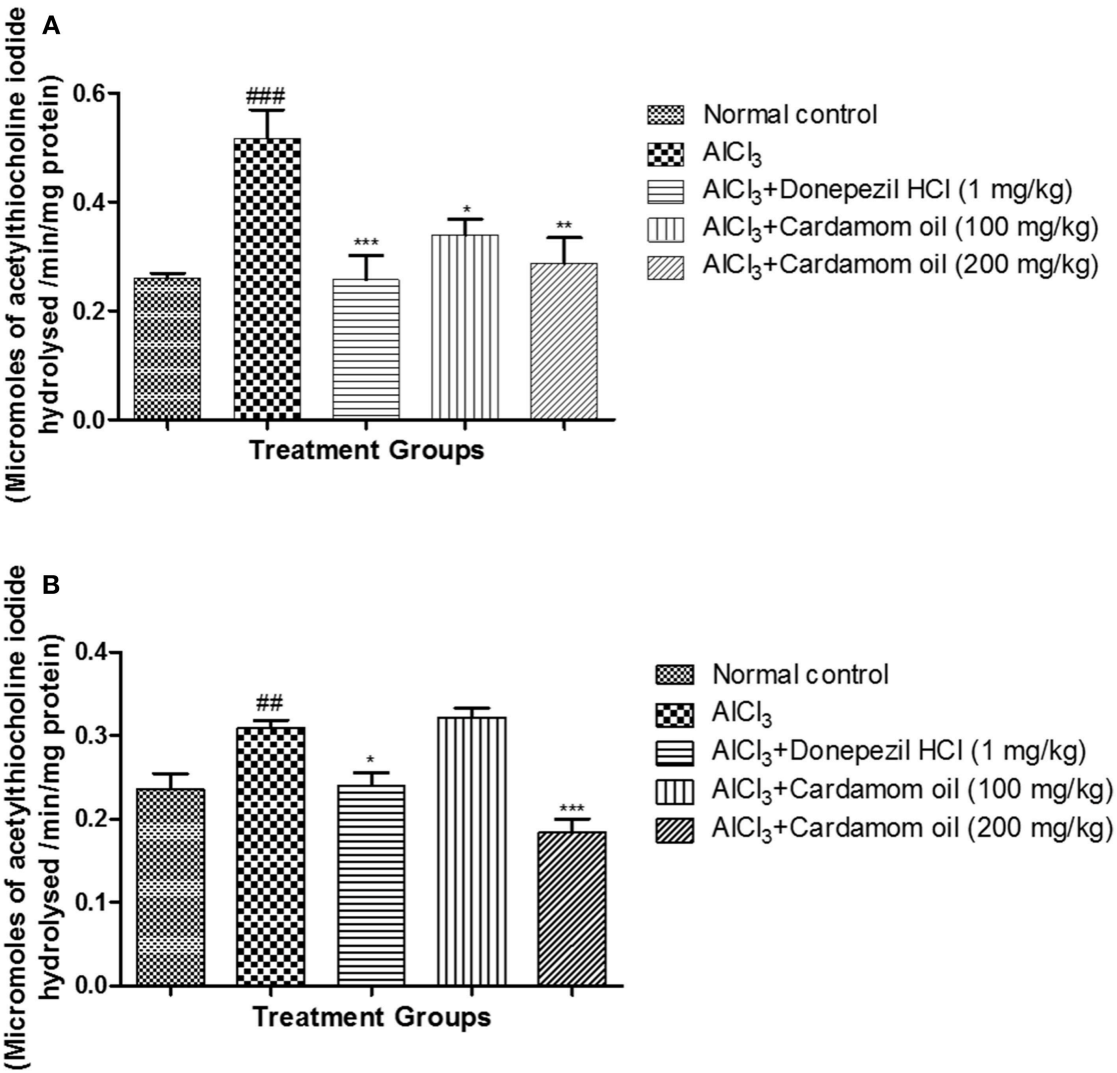
Effect of Cardamom oil on acetylcholinesterase assay in **(A)** hippocampus and **(B)** cortex tissue in AlCl_3_-induced neurotoxicity in rats. Data are expressed as mean ± SEM (*n* = 6), ##p < 0.01, ###*p* < 0.001 when compared with normal control, ^***^p < 0.001, ^**^p < 0.01, ^*^p < 0.05 when compared with disease control group. AlCl_3_, Aluminum chloride; SEM, Standard Error of Mean.

Aluminum chloride treated animals showed a significant increase in AChE activity (*p* < 0.001) in the hippocampus when compared with normal control animals. Treatment with cardamom oil significantly prevented the increase in AChE activity produced by aluminum chloride treatment at a dose of 100 mg/kg (*p* < 0.05) and 200 mg/kg (*p* < 0.01) when compared with disease control animals. The cardamom oil treatment showed comparable results as that of donepezil hydrochloride treated group (Figure 5A). Cortex region showed a significant increase in AChE activity (*p* < 0.01) in aluminum chloride treated animals when compared with normal control animals. Treatment with cardamom oil significantly prevented aluminum chloride induced increase in AChE activity at a dose of 200 mg/kg (*p* < 0.001) when compared with the disease control (Figure 5B).

### Histopathological Studies

#### Hematoxylin and Eosin

**Figure 6:**
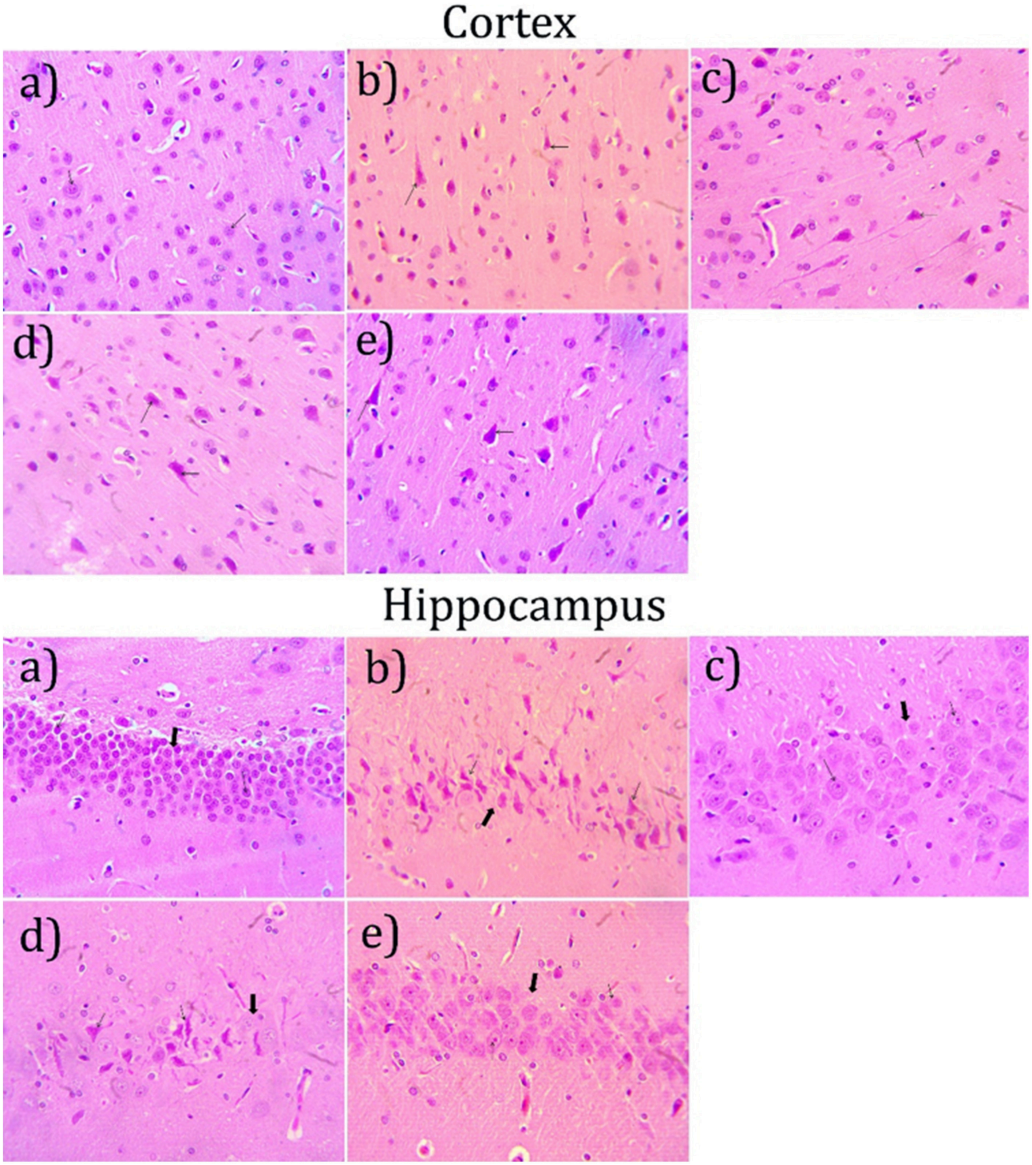
Effect of Cardamom oil on H.E. stained hippocampus and cortex tissue (400X). **(a)** Normal control group: Hippocampus and Cortex: showing normal histology, normal neuronal cell (small arrow) {H & E, 400X}, **(b)** AlCl_3_ group: Hippocampus and Cortex: showing neuronal degeneration with pyknotic nuclei (small arrow), reduced layer of neuronal cell (Large arrow) {H & E, 400X}. **(c)** AlCl_3_ + Donepezil hydrochloride (1 mg/kg): Hippocampus and Cortex: showing normal histology, focal mild neuronal cell with pyknotic nuclei (small arrow) {H & E, 400X}, **(d)** AlCl_3_ + Cardamom oil (100 mg/kg): Hippocampus and Cortex: showing neuronal degeneration with pyknotic nuclei (small arrow), reduced layer of neuronal cell (Large arrow) {H & E, 400X}. **(e)** AlCl_3_ + Cardamom oil (200 mg/kg): Hippocampus and Cortex: showing focal mild neuronal cell with pyknotic nuclei (small arrow) {H & E, 400X}. The magnification was 400X. H.E., Hematoxylin and eosin; AlCl_3_, Aluminum chloride.

Microscopical examination of hippocampus and cortex were observed by staining with hematoxylin and eosin. The disease control group (Figure 6b) showed various histopathological changes like multifocal moderate neuronal degeneration with pyknotic nuclei, multifocal moderate reduced layer of neuronal cell in hippocampus and cortex when compared with normal control group (Figure 6a). Treatment with cardamom oil at a dose of 100 mg/kg (Figure 6d) did not prevent the neuronal degeneration. However, cardamom oil treatment at a dose of 200 mg/kg (Figure 6e) showed a decrease in neuronal degeneration and showed normal histology, normal layer of neuronal cell in when compared with disease control group. The results of cardamom oil treatment at a dose of 200 mg/kg showed comparable results as that of donepezil hydrochloride treated group (Figure 6c).

#### Congo Red Staining

**Figure 7:**
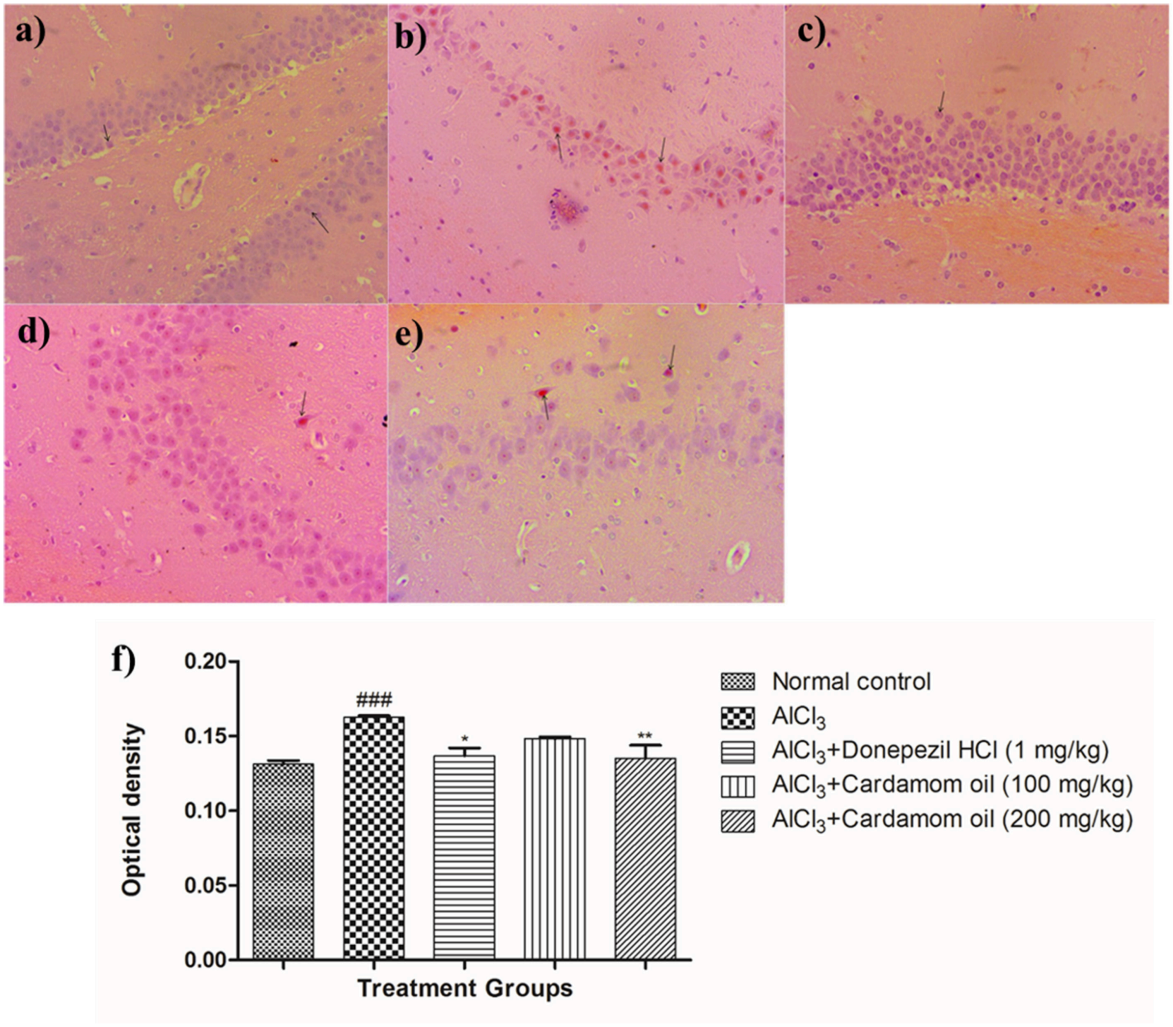
Effect of Cardamom oil on Congo red stained hippocampus tissue (400X). **(a)** Normal control: Hippocampus: showing normal histology, normal neuronal cells in (small arrow) {Congo Red, 400X}, **(b)** AlCl_3_ group: Hippocampus: showing multifocal, moderate deposition of amyloid β plaques (arrow) {Congo Red, 400X}, **(c)** AlCl_3_ + Donepezil hydrochloride (1 mg/kg): Hippocampus: showing normal histology, normal neuronal cells (small arrow) {Congo Red, 400X}, **(d)** AlCl_3_ + Cardamom oil (100 mg/kg): Hippocampus: showing multifocal mild deposition of amyloid β plaques (arrow) {Congo Red, 400X}, **(e)** AlCl_3_ + Cardamom oil (200 mg/kg): Hippocampus: showing multifocal mild deposition of amyloid β plaques (arrow) {Congo Red, 400X}, **(f)** Histogram of congo red staining. Data are expressed as mean ± SEM (*n* = 6), ###*p* < 0.001 when compared with normal control, ^**^p < 0.01, ^*^p < 0.05 when compared with disease control group. The magnification was 400 X. AlCl_3_, Aluminum chloride; SEM, Standard Error of Mean.

**Figure 8:**
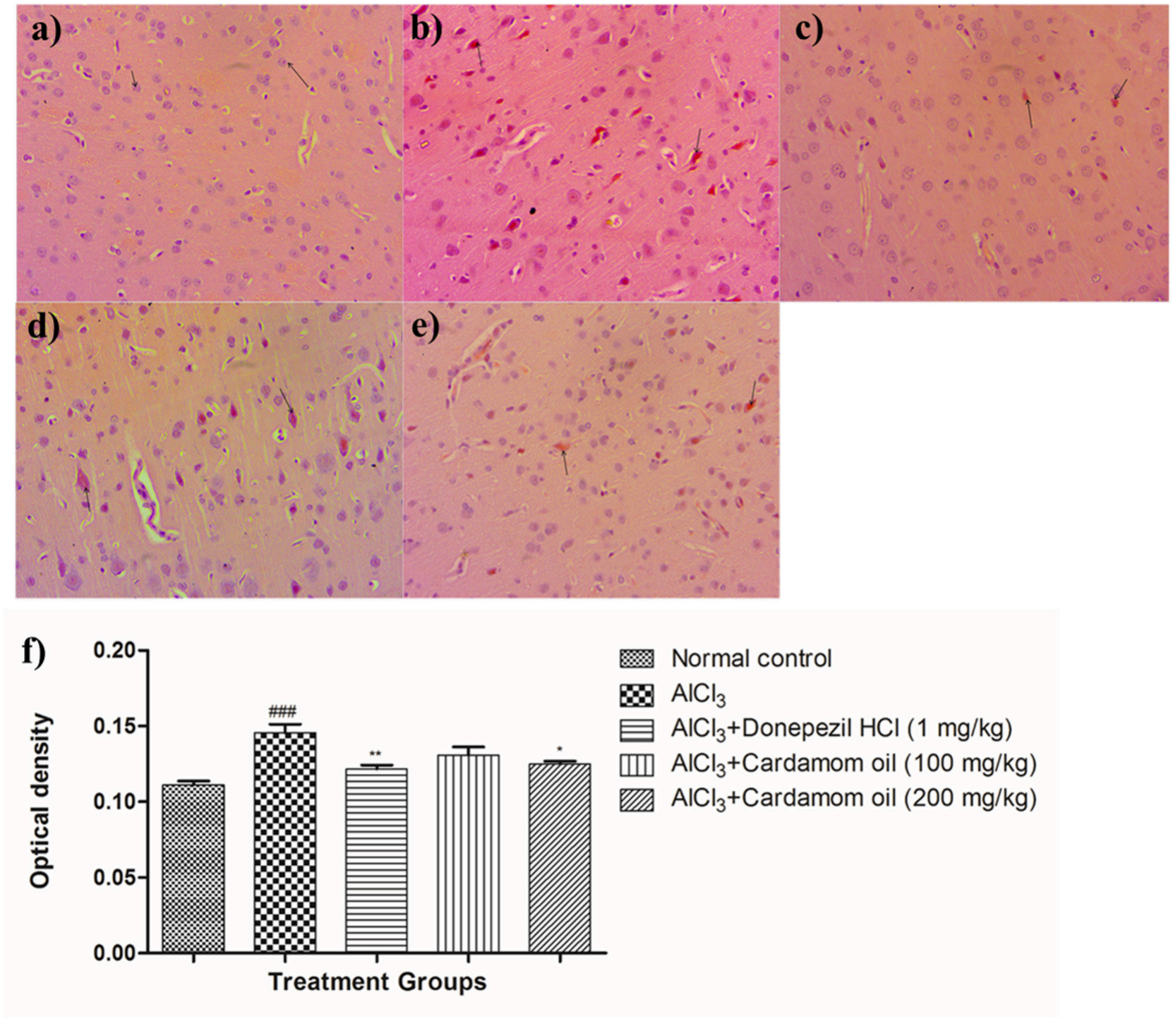
Effect of Cardamom oil on Congo red stained cortex tissue (400X). **(a)** Normal control: Cortex: showing normal histology, normal neuronal cells in (small arrow) {Congo Red, 400X}, **(b)** AlCl_3_ group: Cortex: showing multifocal moderate deposition of amyloid β plaques (arrow) {Congo Red, 400X}, **(c)** AlCl_3_ + Donepezil hydrochloride (1 mg/kg): Cortex: showing multifocal mild deposition of amyloid β plaques (arrow) {Congo Red, 400X}, **(d)** AlCl_3_ + Cardamom oil (100 mg/kg): Cortex: showing multifocal moderate deposition of amyloid β plaques (arrow) {Congo Red, 400X}, **(e)** AlCl_3_ + Cardamom oil (200 mg/kg): Cortex: showing multifocal mild deposition of amyloid β plaques (arrow) {Congo Red, 400X} **(f)** Histogram of congo red staining. Data are expressed as mean ± SEM (*n* = 6), ### *p* < 0.001 when compared with normal control, ^**^p < 0.01, ^*^p < 0.05 when compared with disease control group. The magnification was 400X. AlCl_3_, Aluminum chloride; SEM, Standard Error of Mean.

Hippocampus and cortex were observed by staining with congo red dye to detect the deposition of amyloid beta plaques. The disease control animals (Figures 7b, 8b) showed multifocal moderate deposition of amyloid beta plaques at hippocampus and cortex when compared with normal control animals (Figures 7a, 8a). Treatment with cardamom oil at a dose of 100 mg/kg (Figures 7d, 8d) did not decrease the deposition of amyloid beta plaques to normal and showed mild to moderate plaque lesions in hippocampus and cortex region. At a dose of 200 mg/kg of cardamom oil (Figures 7e, 8e) treatment showed a decrease in the deposition of amyloid beta plaques and observed intact, normal histology in hippocampus and cortex when compared with disease control animals. These results are comparable to that of donepezil hydrochloride treated group (Figures 7c, 8c). The optical density data of congo red stained sections has been provided in Figures 7f, 8f.

### Amyloid β Expression in Brain Tissue by Using Immunohistochemistry Studies (IHC)

**Figure 9:**
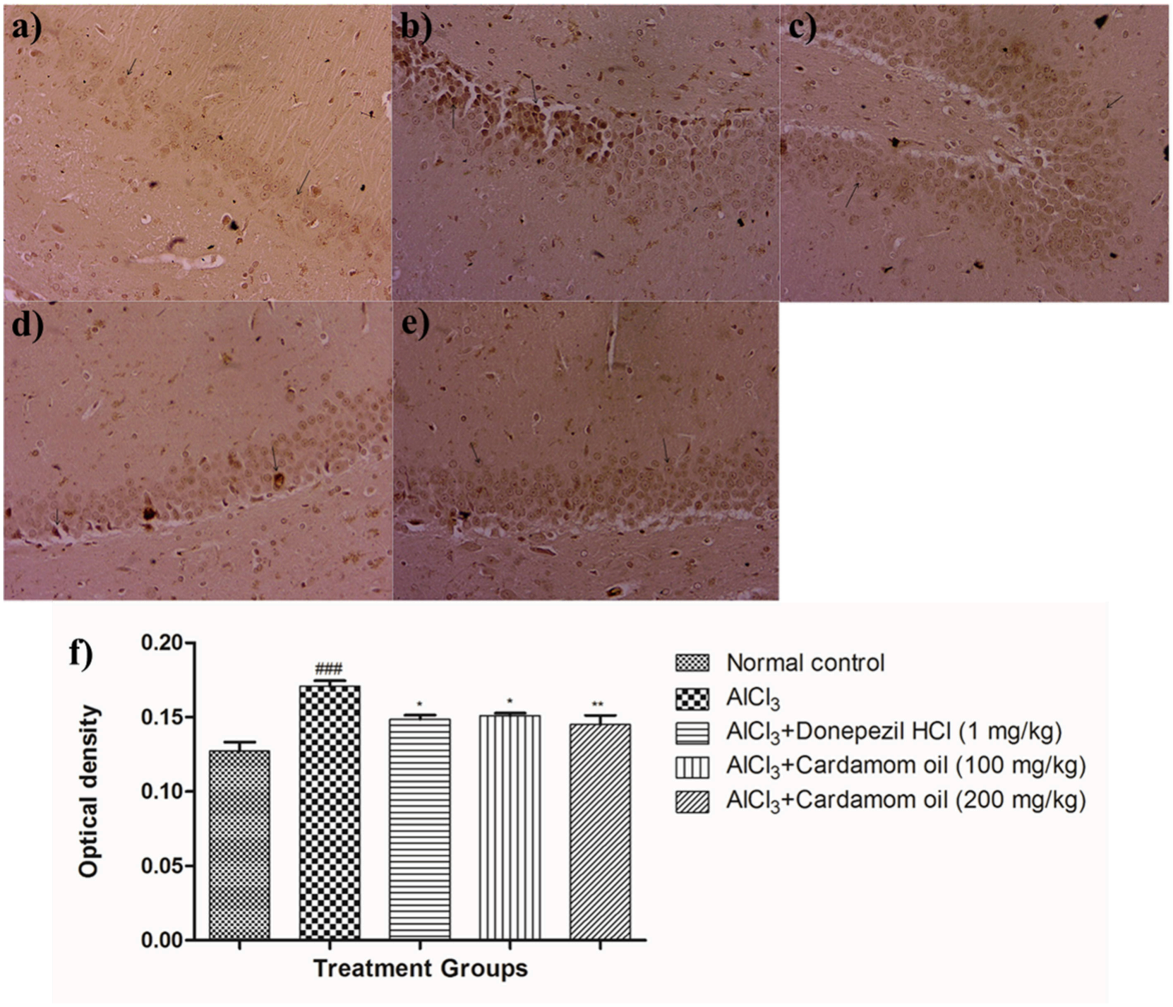
Effect of Cardamom oil on amyloid β expression in hippocampus tissue (400X). **(a)** Normal control: Hippocampus: showing normal neuronal cells without immunoreactivity (arrow){Immunostaining: Amyloid- β, 400X}, **(b)** AlCl_3_ group: Hippocampus: showing significantly mild enhanced expression of amyloid- β, exhibited by brown coloration (arrow){Immunostaining: Amyloid- β, 400X}, **(c)** AlCl_3_ + Donepezil hydrochloride (1 mg/kg): Hippocampus: showing normal histology, normal neuronal cells without immunoreactivity (arrow) {Immunostaining: Amyloid- β, 400X}, **(d)** AlCl_3_ + Cardamom oil (100 mg/kg): Hippocampus: showing significantly enhanced expression of amyloid- β, exhibited by brown coloration (arrow) {Immunostaining: Amyloid- β, 400X}, **(e)** AlCl_3_ + Cardamom oil (200 mg/kg): Hippocampus: showing normal histology, normal neuronal cells without immunoreactivity (arrow){Immunostaining: Amyloid- β, 400X}, **(f)** Histogram of IHC. Data are expressed as mean ± SEM (*n* = 6), ###*p* < 0.001 when compared with normal control, ^**^p < 0.01, ^*^p < 0.05 when compared with disease control group. The magnification was 400X. AlCl_3_, Aluminum chloride; SEM, Standard Error of Mean; IHC, Immunohistochemistry.

**Figure 10:**
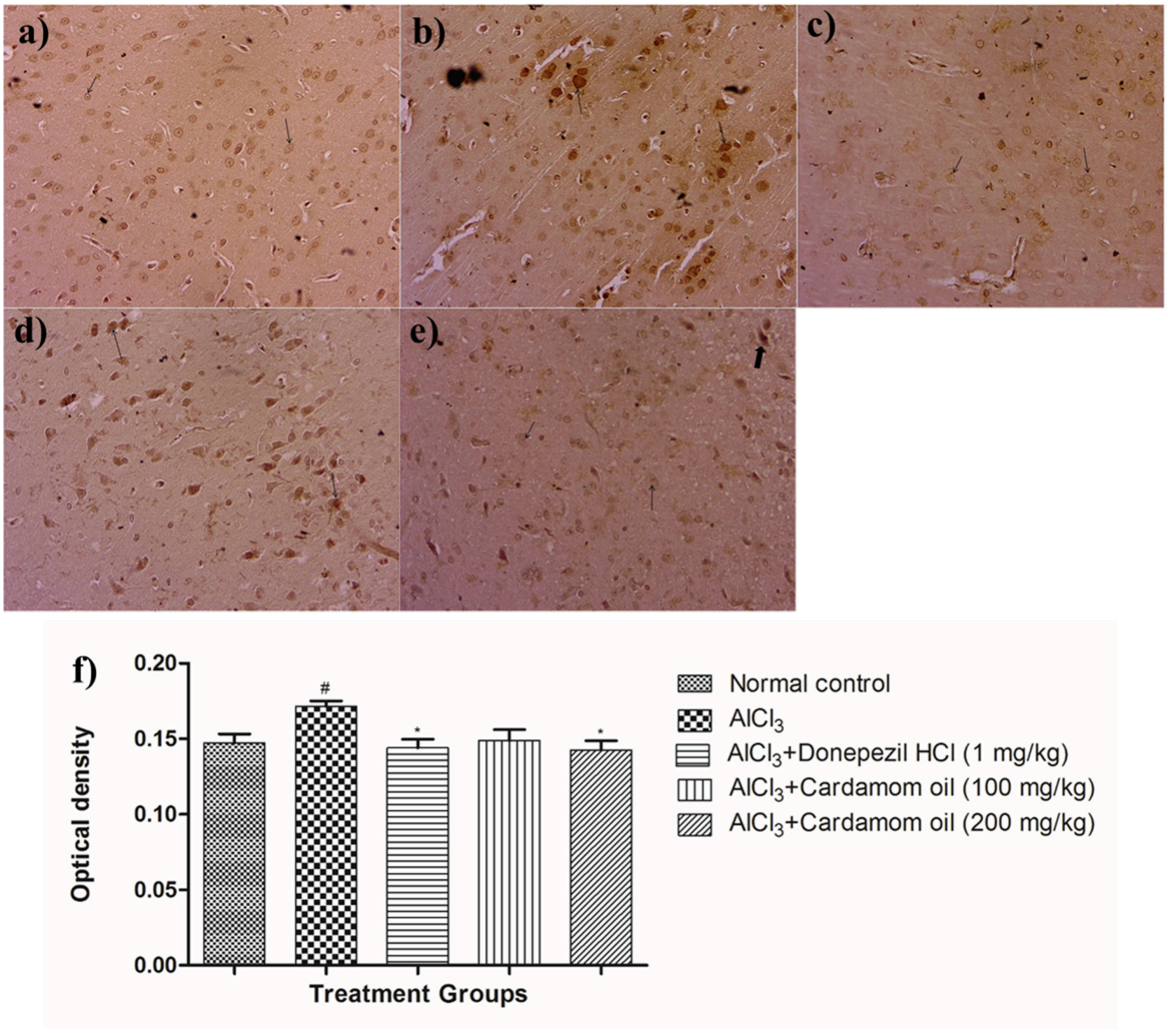
Effect of cardamom oil on amyloid β expression in cortex tissue (400X). **(a)** Normal control: Cortex: showing normal neuronal cells without immunoreactivity (arrow){Immunostaining: Amyloid- β, 400X}, **(b)** AlCl_3_ group: Cortex: showing significantly enhanced expression of amyloid- β, exhibited by brown coloration at cortical neurons {Immunostaining: Amyloid- β, 400X}, **(c)** AlCl_3_ + Donepezil hydrochloride (1 mg/kg): Cortex: showing normal histology, normal neuronal cells without immunoreactivity (arrow) {Immunostaining: Amyloid- β, 400X}, **(d)** AlCl_3_ + Cardamom oil (100 mg/kg): Cortex: showing normal histology, significantly enhanced expression of amyloid- β, exhibited by brown coloration at cortical neurons (arrow) {Immunostaining: Amyloid- β, 400X}, **(e)** AlCl_3_ + Cardamom oil (200 mg/kg): Cortex: showing normal neuronal cells without immunoreactivity at cortex (small arrow), note weak immunoreactivity (large arrow){Immunostaining: Amyloid- β, 400X}, **(f)** Histogram of IHC. Data are expressed as mean ± SEM (*n* = 6), #*p* < 0.05 when compared with normal control, ^*^p < 0.05 when compared with disease control group. The magnification was 400 X. AlCl_3_, Aluminum chloride; SEM, Standard Error of Mean; IHC, Immunohistochemistry.

Immunohistochemical analysis of hippocampus and cortex region showed significantly increased the expression of amyloid- β in the disease control animals (Figures 9b, 10b) while very weak expression was observed in normal control animals (Figures 9a, 10a). Optical density (OD) of the disease control group was significantly higher as compared with that of normal control animals (Figures 9f, 10f). Treatment with cardamom oil at a dose of 100 mg/kg (Figures 9d, 10d) did not decrease expression of amyloid- β in hippocampus and cortex to normal. However, treatment with cardamom oil at a dose of 200 mg/kg (Figures 9e, 10e) showed a decrease in amyloid- β expression and showed normal histology, weak expression in hippocampus and cortex when compared with disease control animals. The results at a dose of 200 mg/kg of cardamom oil are comparable as that of donepezil hydrochloride treated group (Figures 9c, 10c).

### BDNF Expression in Brain Tissue by Using Immunohistochemistry Studies (IHC)

**Figure 11:**
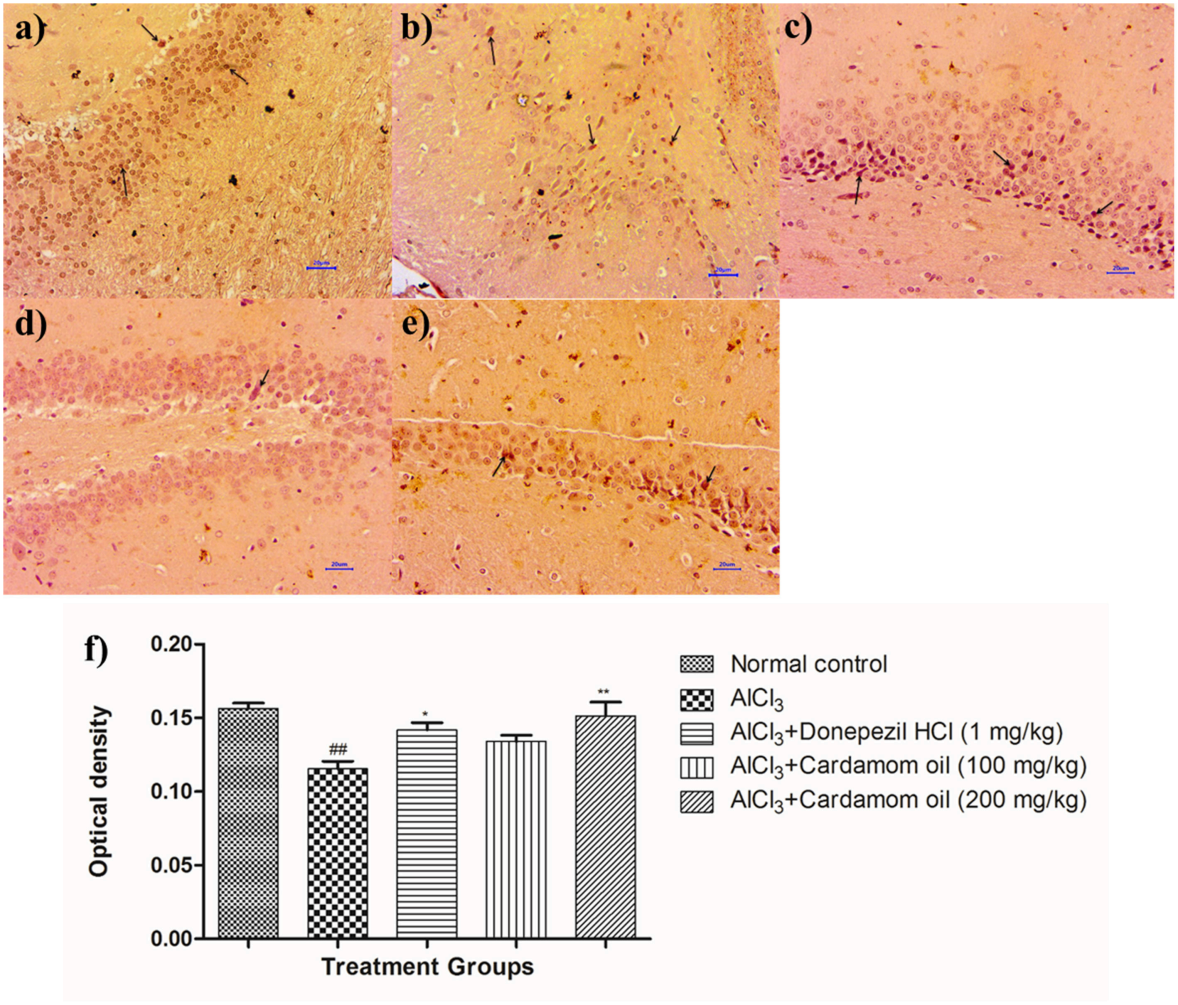
Effect of cardamom oil on BDNF expression in hippocampus tissue (400X). **(a)** Normal control: Hippocampus: showing mild enhanced expression of BDNF, exhibited by brown coloration at hippocampal neurons (arrow) {Immunostaining: BDNF, 400X}, **(b)** AlCl_3_ group: Hippocampus: showing reduced expression of BDNF, exhibited by brown coloration at hippocampal neurons (arrow) {Immunostaining: BDNF, 400X}, **(c)** AlCl_3_ + Donepezil hydrochloride (1 mg/kg): Hippocampus: showing moderately enhanced expression of BDNF, exhibited by brown coloration at hippocampal neurons (arrow) {Immunostaining: BDNF, 400X}, **(d)** AlCl_3_ + Cardamom oil (100 mg/kg): Hippocampus: showing reduced expression of BDNF, exhibited by brown coloration at hippocampal neurons (arrow) {Immunostaining: BDNF, 400X}, **(e)** AlCl_3_ + Cardamom oil (200 mg/kg): Hippocampus: showing normal histology, mild enhanced expression of BDNF, exhibited by brown coloration at hippocampal neurons (arrow) {Immunostaining: BDNF, 400X}, **(f)** Histogram of IHC. Data are expressed as mean ± SEM (*n* = 6), ##*p* < 0.01 when compared with normal control, ^**^p < 0.01, ^*^p < 0.05 when compared with disease control group. The magnification was 400X. AlCl_3_, Aluminum chloride; SEM, Standard Error of Mean; BDNF, brain-derived neurotrophic factor; IHC, Immunohistochemistry.

**Figure 12:**
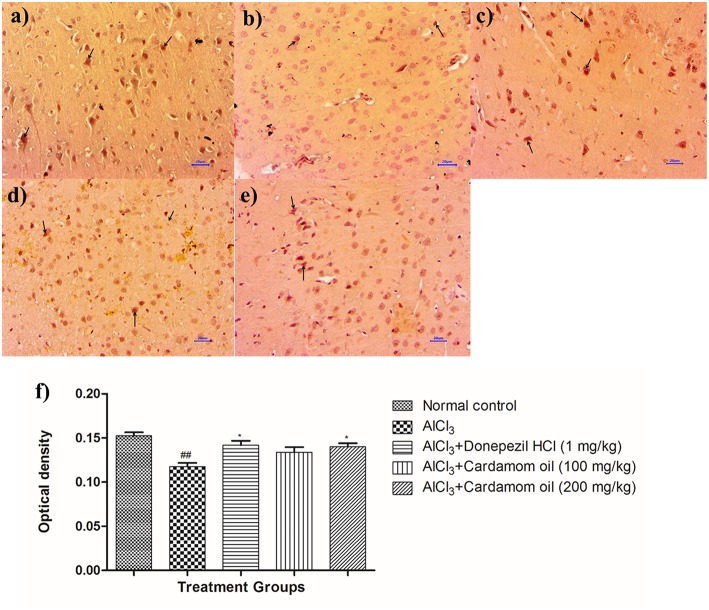
Effect of cardamom oil on BDNF expression in cortex tissue (400X). **(a)** Normal control: Cortex: showing moderately enhanced expression of BDNF, exhibited by brown coloration at cortical neurons (arrow) {Immunostaining: BDNF, 400X}, **(b)** AlCl_3_ group: Cortex: showing reduced expression of BDNF, exhibited by brown coloration at cortical neurons (arrow) {Immunostaining: BDNF, 400X}, **(c)** AlCl_3_ + Donepezil hydrochloride (1 mg/kg): Cortex: showing moderately enhanced expression of BDNF, exhibited by brown coloration at cortical neurons (arrow) {Immunostaining: BDNF, 400X}, **(d)** AlCl_3_ + Cardamom oil (100 mg/kg): Cortex: showing reduced expression of BDNF, exhibited by brown coloration at cortical neurons (arrow) {Immunostaining: BDNF, 400X}, **(e)** AlCl_3_ + Cardamom oil (200 mg/kg): Cortex: showing mild enhanced expression of BDNF, exhibited by brown coloration at cortical neurons (arrow) {Immunostaining: BDNF, 400X,}, **(f)** Histogram of IHC. Data are expressed as mean ± SEM (*n* = 6), ##*p* < 0.01 when compared with normal control, ^*^p < 0.05 when compared with disease control group. The magnification was 400X. AlCl_3_, Aluminum chloride; SEM, Standard Error of Mean; BDNF, brain- derived neurotrophic factor; IHC, Immunohistochemistry.

Immunohistochemical analysis of hippocampus and cortex region showed significantly decreased the BDNF expression in disease control animals (Figures 11b, 12b) as compared to normal control animals (Figures 11a, 12a). Optical density (OD) of disease control group was significantly lower as compared with that of normal control animals (Figures 11f, 12f). Treatment with cardamom oil at a dose of 100 mg/kg (Figures 11d, 12d) did not increase the expression of BDNF in hippocampus and cortex to normal. However, treatment with cardamom oil at a dose of 200 mg/kg (Figures 11e, 12e) showed the increase in BDNF expression in hippocampus and cortex when compared with disease control animals. The results at a dose of 200 mg/kg of cardamom oil are comparable as that of donepezil hydrochloride treated group (Figures 11c, 12c).

### Human Equivalent Dose

In present study, optimum dose of cardamom oil in rats was found to be 200 mg/kg.

$$\begin{array}{l} \text{Human Equivalent Dose }\left(\text{HED}\right)\\ \text{of cardamom oil }\left(\text{mg}/\text{kg}\right)=200\text{ mg}/\text{kg}\times 0.16\\ \;=32\text{mg}/\text{kg}\\ \text{ Human dose }\left(70\text{kg}\right)=2.24\text{gm}/\text{day} \end{array}$$

Thus, dose of cardamom oil upto 2.24 gm/day can be given in 70 kg human.

## Discussion

Several genetical and environmental factors are involved in etiology of Alzheimer's disease. Genetic mutation related to both metabolism and expression of amyloid precursor protein (APP) are considered to be important for diagnosis of familial Alzheimer's disease (50). In addition, exposure to environmental metals for long time instigate neurodegenerative disorders, including Alzheimer's disease (51). Aluminum (Al) exposure occurs mainly through occupational, environment and dietary factors for humans (52). Aluminum has a cholinotoxin nature which causes apoptotic neuronal loss that lead to neurodegeneration associated with AD (6). Aluminum is non-redox trivalent cation and has been recognized as a causative factor in various neurological disorders due to its neurotoxicity (53). Accumulation of aluminum in the brain has been reported to be one of the contributing factor in AD, where aluminum affects integrity and permeability of blood-brain barrier (BBB) by altering the lipophilic characteristics of the same.(54, 55). Deposition of amyloid beta, hyperphosphorylation of tau protein (52, 56), increase in AChE activity (5), imbalance in level of neurotransmitters, inflammatory cytokines (12), memory and learning deficits (57) are important manifestations caused due to aluminum neurotoxicity, which is involved in the etiology of AD (58). Also mitochondrial damage and oxidative stress-induced lipid peroxidation are strongly associated with neuronal cell death, which is observed in Alzheimer's disease (58, 59). So, we have selected animal model of aluminum chloride induced Alzheimer's disease in the present study.

The GC-MS analysis showed the main constituent of cardamom oil is 1,8 Cineole (31.09%). Study reported that 1,8 cineole inhibits AChE *in vitro* (60), improves cognition (61) and reported to have an anti-inflammatory action (32) in neurodegenerative disease like AD. So it has been proposed that treatment with 1,8 cineole rich cardamom oil can be useful and one of the important alternative in pharmacotherapy of AD. The results of present study showed that AlCl_3_ administration resulted into progressive deterioration of spatial and recognition memory in rats. EPM test is used for determination of spatial learning and memory by measuring initial transfer latency (ITL) and retention transfer latency. In this test, decrease in RTL after ITL determination indicated improvement in recognition memory of rats. Cardamom oil treatment significantly reduced RTL of rats as compared to aluminum chloride treated group. MWM test is used to evaluate cognitive functions related to spatial learning by measuring escape latency (EL). EL is the time required to locate the hidden submerged platform. Decrease in EL indicated improvement in spatial learning and memory of rats. In this study, cardamom oil administration significantly decreased EL of rats as compared to aluminum chloride treated group indicating improvement in learning and memory skills. Accumulation of aluminum in the brain interfere with long term potentiating molecule such as cyclic GMP which leads to an impairment in glutamate-NO-cGMP pathway that results into cognitive impairments and neurobehavioral deficits in animals (62). Cardamom oil treatment reversed the spatial cognitive impairment. PA is a fear-motivated test used to evaluate short term and long term memory by recording pre and post-shock latencies. An increased in post-shock latency was observed in rats treated with cardamom oil as compared to the aluminum chloride treated group. In this test, the increase in post-shock latency after the pre-shock latency indicated improvement in recognition memory of rats. In present study, aluminum chloride treatment showed reduction in retention latency due to anxiogenic-like behavior in animals, cardamom oil treatment showed increase in retention latency. This activity may be due to neuroprotective effect of cardamom oil. Substantial locomotion impairment was observed in disease rats; previous literature reports of chronic AlCl_3_ administration have been shown, a decrease in locomotion activity as well, which is implicated to be a central nervous system depression (63). Treatment with cardamom oil improved locomotor activity and behavioral impairments in rats.

Acetylcholinesterase (AChE) is the key enzyme involved in the hydrolysis of the acetylcholine in the neuromuscular junction and synapse which results in termination of the nerve impulse transmission (64). The impairments in cholinergic transmission are associated with the severity of AD (65). High level of AChE is found in the brain of AD patients (66). Previous reports suggested that activity of the AChE increases, which lead to a decrease in acetylcholine level in the brain of AD patients (67). Neurotoxic effect of aluminum significantly increases AChE activity which is responsible for hydrolysis of acetylcholine (54). The increase in activity of AChE by aluminum could be due to the allosteric interaction between cation (Al^+3^) and anionic sites of acetylcholinesterase enzyme which lead to alteration in secondary structure of AChE in the brain (5). Inhibition of acetyl-cholinesterase (AChE) serves as an important strategy in the development of anti-Alzheimer's drugs. Natural products like essential oils have been reported for their strong acetylcholinesterase (AChE) inhibitory activity (21, 22, 68). In our study, increased in AChE activity was observed in aluminum chloride treated group when compared to normal control rats. Treatment with cardamom oil showed decrease in AChE activity significantly, due to its main chemical constituent 1,8 cineole which has been reported to inhibit AChE enzyme *in vitro* (27, 32, 60).

Lipid peroxidation is one of the important biomarker of oxidative stress. It has been reported that aluminum activates and stimulates lipid peroxidation in presence of iron in the brain (69). Aluminum increases iron-induced oxidative injury by enhancing iron-based oxidation in the brain that alters iron homeostasis mainly via the Fenton reaction (53, 70). Recently it has been reported that aluminum is causitive factor for modulation in the brain amyloidosis through oxidative damage (71). As increased in oxidative stress lead to inhibition of various endogenous antioxidant enzymes such as catalase, superoxide dismutase, reduced glutathione due to redox reaction which have crucial role against free radical damage (72). In our study, aluminum chloride treatment showed marked increase in oxidative stress in the brain which was indicated by decrease in level of catalase, superoxide dismutase, reduced glutathione, and increase in malondialdehyde (MDA) level leading to neuronal damage (73, 74). In the present study, treatment with cardamom oil reduced oxidative damage by increasing catalase, superoxide dismutase, and reduced glutathione levels and by decreasing MDA level.

Hematoxylin and eosin-stained brain tissue showed increased neurodegeneration with pyknotic nuclei observed in disease control group. Cardamom oil treatment ameliorated these degenerative changes in the hippocampus and cortex region of the brain due to its neuroprotective action. It has been reported that aggregation and deposition of amyloid beta (Aβ) are the main culprits in the pathogenesis of AD which leads to neurodegeneration (75). Also, AChE promotes the aggregation of amyloid beta plaques and neurofibrillary tangles, which are pathological features of AD (7, 76). Deposition of Aβ plaques was assessed by using congo red dye (45). It has been reported that accumulation of aluminum in the brain is responsible for the formation and deposition of amyloid beta protein (71). Multifocal moderate deposition of Aβ plaques observed in disease control group. However, treatment with cardamom oil showed a decrease in deposition of Aβ plaques in hippocampus and cortex region. In Immunohistochemistry (IHC) analysis expression of Aβ significantly increased in the disease control group. In present study, treatment with cardamom oil showed significant reduction in Aβ expression in hippocampus and cortex region of the brain. It has been reported that expression of BDNF decreased in patients with Alzheimer's disease (77, 78). *In vitro* and *in vivo* studies has been reported that BDNF enhances central cholinergic neurotransmission. (77). It has been also reported that BDNF regulates and modulates synaptic neuronal plasticity which results into increase in synaptic transmission and shows neuroprotective effect in the brain insults (10, 79). In present study, treatment with cardamom oil showed significant increase in BDNF expression in hippocampus and cortex region of the brain.

## Conclusion

Cardamom oil treatment produced improvement in the neurobehavioral parameters like cognitive functions and anti-anxiety effect. It also inhibited acetylcholinesterase activity in hippocampus and cortex and improved glutathione, catalase and superoxide dismutase, lipid peroxidation levels in the brain indicating reduction in oxidative damage. Treatment with cardamom oil also reduced neuronal degeneration by increasing BDNF level and inhibiting amyloid β expression in hippocampus and cortex. The formation of amyloid β plaque was inhibited by cardamom oil treatment. Cardamom oil showed a neuroprotective effect via inhibition of AChE activity and reduction in oxidative stress. As there are limited approaches for AD management; cardamom oil may provide a safe, economic and therapeutic alternative in the management of Alzheimer's disease.

## Ethics Statement

The study was carried out in accordance with CPCSEA guidelines. The protocol was approved by the Institutional Animal Ethics Committee (approval no. CPCSEA/IAEC/P-24/2018).

## Author Contributions

YK has designed the study. SA carried out the experimental work. YK and SA have written the manuscript.

### Conflict of Interest Statement

The authors declare that the research was conducted in the absence of any commercial or financial relationships that could be construed as a potential conflict of interest.

## Abbreviations

AD: Alzheimer's disease
AChE: acetylcholinesterase
Aβ: amyloid beta
BBB: blood-brain barrier
BDNF: brain-derived neurotrophic factor
DTNB: dithio-bis nitrobenzoic acid
DPX: digital Picture Exchange
DAB: diaminobenzidine
IHC: immunohistochemistry
MDA: malondialdehyde
NMDA: N-methyl-D-aspartate
NO-cGMP: nitric oxide-cyclic guanosine monophosphate
SOD: superoxide dismutase.
